# Comparative Nutritional Profile of Publicly Procured Foods for School Meals in Federal Schools in Northeastern Brazil Pre- and Post-COVID-19 Pandemic

**DOI:** 10.3390/nu17010134

**Published:** 2024-12-31

**Authors:** Wilma Fabiana Ferreira da Silva, Ingrid Wilza Leal Bezerra, Diogo Vale, Antonio Gouveia Oliveira, Larissa Mont’Alverne Jucá Seabra

**Affiliations:** 1 Post-Graduation Program in Nutrition, Federal University of Rio Grande do Norte, Natal 59078-900, RN, Brazil; fabianaferreiranutri@gmail.com; 2 Department of Nutrition, Federal University of Rio Grande do Norte, Natal 59078-900, RN, Brazil; ingrid.bezerra@ufrn.br; 3 Federal Institute of Education, Science and Technology of Rio Grande do Norte, Natal 59015-000, RN, Brazil; diogovaleufrn@gmail.com; 4 Pharmacy Department, Health Sciences Center, Federal University of Rio Grande do Norte, Natal 59078-970, RN, Brazil; antonio.gouveia@ufrn.br

**Keywords:** school feeding, nutritive value, food labeling, competitive bidding, COVID-19

## Abstract

School meals play a critical role in supporting students’ biopsychosocial growth, development, learning, academic performance, and the establishment of healthy eating habits. In public institutions, food procurement is conducted through formal public procurement processes. However, emphasizing cost-effectiveness in bidding criteria, such as prioritizing the lowest product price, may inadvertently encourage the acquisition of foods high in critical nutrients. In Brazil, specific guidelines for public school food procurement have been introduced to improve the nutritional quality of foods included in school menus. Objective: To evaluate the impact of legislative measures and regulations implemented during the COVID-19 pandemic on the nutritional composition of school meals provided by federal high schools in Rio Grande do Norte, located in northeastern Brazil. It also compared the composition and origin of foods procured before and after the pandemic. Methods: This is a longitudinal observational panel study conducted across 20 federal schools. Procurement documents from 2019 (pre-pandemic) and 2021 (post-pandemic) were analyzed to assess changes in the nutritional profile of procured foods. Results: Post-pandemic, the procurement of natural and minimally processed and processed foods increased, representing 73.39% and 10.34%, respectively, of the total approved foods. There was also a 39% reduction in ultra-processed foods compared to 2019, in addition to a reduction in culinary ingredients. Additionally, the proportion of foods containing excessive levels of critical nutrients declined. A significant shift was observed in the origin of procured foods, with a notable increase in the purchase of locally sourced items. Conclusions: The findings indicate a positive shift in the nutritional quality of foods procured after the COVID-19 pandemic, with a decrease in ultra-processed food purchases and an increase in the procurement of natural and minimally processed options. Nonetheless, the continued presence of ultra-processed foods and items with excessive critical nutrients highlights the need for further improvements in public procurement practices to fully align with nutritional guidelines.

## 1. Introduction

School feeding (SF) is one of the most important public policies for food and nutrition security in the world [1,2]. In Brazil, the National School Feeding Program (PNAE) aims to develop students in terms of learning, growth, biopsychosocial development, academic performance, and the formation of healthy eating habits, in addition to promoting meals that are compatible with the student’s energy and nutritional needs [1,2,3,4]. The PNAE also contributes to the development of the local economy through policies to encourage family farming, which determine a minimum of 30% of financial resources allocated to purchasing food from this source [1,2,5].

Given the nutritional epidemiological reality that exposes the growing number of overweight and obese children and adolescents, the World Health Organization and the Pan American Health Organization (PAHO) jointly developed, in 2014, the Plan of Action for the Prevention of Obesity in Children and Adolescents. One of its key objectives is to enhance the quality of meals provided in schools [2]. As part of this strategy, in 2016, the PAHO introduced the Nutrient Profile Model. This model establishes specific criteria for assessing processed and ultra-processed foods based on their content of critical nutrients, including sodium, free sugars, sweeteners, total fats, saturated fats, and trans fats [6].

However, despite the implementation of numerous public policies related to SF, as well as a substantial body of documents and studies published on this subject, several barriers still hinder the provision of adequate healthy food in the school environment. One of the most significant barriers is the food procurement process [1,2,7,8]. In Brazil, the food purchasing model for public institutions is conducted through the public procurement process, specifically using the electronic competitive bidding modality, which is carried out remotely via the Federal Government’s procurement system. This modality is governed by normative acts that promote fair competition among suppliers [9], but it often introduces bureaucratic challenges into the procurement cycle. Thus, despite various legislative measures intended to facilitate the acquisition of healthy foods through Public Calls (a mechanism within Brazil’s procurement framework designed to prioritize purchasing food from family farming) for purchasing food from family farming, a considerable presence of ultra-processed foods in school meals is still observed [1,3,4,7].

Purchasing through public procurement also highlights challenges related to the origin of foods. A significant portion of the inputs used in public school meals are produced by industries concentrated in the southeastern and southern regions of Brazil, often traveling long distances to reach other parts of the country. This results in an extensive supply chain from food production to consumption, generating social and environmental impacts that counter efforts to promote local consumption and the objectives of Sustainable Development Goal (SDG) 12, which advocates for promoting sustainable public procurement practices [10,11].

Some changes were made to the guidelines for implementing the PNAE between 2020 and 2021, particularly with the publication of a new resolution in 2020 [4], which introduced updated parameters for food procurement and menu planning in school meals. One of the main changes was the limited procurement of ultra-processed foods. The COVID-19 pandemic prompted the publication of additional regulations to adapt the implementation of the PNAE during this period. The World Health Organization declared COVID-19 a pandemic on 11 March 2020. In Brazil, the first cases were confirmed in February 2020, leading to the declaration of a Public Health Emergency of National Importance and a nationwide quarantine in March 2020. This resulted in the suspension of classes, the closure of businesses, and various other measures to mitigate the impacts of the pandemic [12].

Thus, following the critical situation at the time, which led to the suspension of in-person classes, foodstuffs were distributed to the guardians of public school students using resources from the PNAE. This initiative aimed to mitigate the effects of food insecurity resulting from the pandemic [13]. It was stipulated that the foods be distributed in kits defined by the local nutrition team [14]. Even after the pandemic, the social consequences of this time continued to affect the implementation of PNAE, introducing further obstacles to the public procurement process and, consequently, influencing the acquisition of school meal foods [15].

Therefore, the aim of this study was to assess whether legislative regulations implemented during the COVID-19 pandemic decreased the offering of processed and ultra-processed foods and beverages containing an excess of critical nutrients as defined by PAHO (sugars, sodium, total fat, saturated fat, and trans fat) in school meals provided by federal high schools in the state of Rio Grande do Norte in northeastern Brazil. Specifically, this study compared the foodstuffs purchased before and after the pandemic and the origin of the purchased foods.

## 2. Materials and Methods

### 2.1. Study Characterization

This is a longitudinal observational panel study based on document analysis to evaluate food items publicly procured through electronic competitive bidding in federal high schools. As the data used in this study is publicly accessible, approval from the Research Ethics Committee was not required. This study included all 20 schools of the Federal Institute of Rio Grande do Norte (IFRN) offering integrated technical and high school education, encompassing approximately 17,000 beneficiaries of the PNAE [15].

### 2.2. Data Collection

Data on foods procured for school meals before and after the COVID-19 pandemic (2019 and 2021) were obtained from the official federal government procurement platform (comprasnet.gov.br, accessed on 1 September 2022) by consulting fiscal documents related to purchases made through the public procurement modality. The respective electronic competitive bidding processes and their minutes included details such as the description of the foods, specifications, brands, suppliers, and producers. Foods were considered procured if approved in the analyzed competitive bidding processes and made available for acquisition by the schools.

### 2.3. Nutritional Profile Assessment

First, all foods procured in 2019 and 2021 were identified. These foods were then categorized according to the NOVA classification, which divides foods into four categories based on the extent and purpose of their processing: unprocessed or minimally processed (UN), processed (PR), ultra-processed (UP), and culinary ingredients (CI) [16,17,18].

Processed and ultra-processed foods were assessed for their nutritional profile using the PAHO Nutrient Profile Model [6]. This evaluation considered excessive levels of sodium (sodium density in mg/kcal ≥ 1), free sugars (energy density ≥ 10%), total fats (energy density ≥ 30%), saturated fats (energy density ≥ 10%), trans fats (energy density ≥ 1%), and the presence of sweeteners (either artificial or natural, caloric, or non-caloric). Nutritional information was obtained from the food labels available on the official websites of the brands, in local supermarkets, or, when the original labels could not be accessed, from the Brazilian Food Composition Table (TBCA) [19]. Additionally, foods based on Genetically Modified Organisms (GMOs) were identified by checking for the presence of the “T” symbol on product packaging [20].

### 2.4. Food Origin

The origin of packaged foods was determined based on information provided on labels or by identifying the producer’s location. For unpackaged products, such as fresh fruits and vegetables, the supplier’s origin was verified through the location of the company responsible for providing the food. Three categories of origin were considered: local, if the origin was within the state of RN; regional, when the food originated in another state within the northeastern region of Brazil; and national if the food was sourced from other regions in Brazil.

### 2.5. Statistical Analysis

For the classification of processing levels, the number of items procured by each institution was considered, regardless of how many times the same item was procured. For the analysis of excess critical nutrients, the types of products procured by each institution were considered, distinguishing between products when the same item was associated with different brands. All items listed in the procurement process were considered for identifying the origin of foods and classifying the level of processing.

The level of processing and nutritional profile of foods procured in the pre- and post-COVID periods were compared using two-tailed paired *t*-tests, with differences considered statistically significant at *p* < 0.05. Differences in the number of procurements related to the origin of foods between the pre- and post-COVID periods were analyzed using an interaction test between product origin (local, regional, national) and year (2019, 2021) through mixed-effects linear regression, with the school as a random effect and origin and year as fixed effects. Paired t-tests were conducted for comparisons between 2019 and 2021 for each origin category. Statistical analyses were performed using Stata 15 software (Stata Corp., College Station, TX, USA).

## 3. Results

### 3.1. Level of Processing of Publicly Procured Foods

In 2019 and 2021, four procurement processes were conducted for the federal schools studied (two in 2019 and two in 2021), resulting in the approval of 384 food items in 2019 and 387 in 2021. The food items approved in these procurement processes are categorized into nine food groups, as shown in Table 1 below.

**Figure 1:**
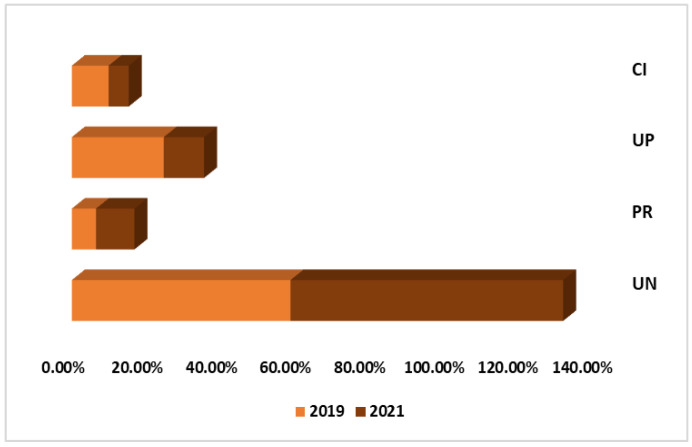
Percentage of foods publicly procured by NOVA classification in Federal Schools of Rio Grande do Norte in 2019 and 2021. CI: culinary ingredients; UP: ultra-processed; PR: processed; UN: unprocessed or minimally processed.

Figure 1 presents the percentage of foods procured by NOVA classification in 2019 and 2021. In 2019, 58.85% of the food procured belonged to the UN group, 6.51% to the PR group, 24.74% to the UP group, and 9.90% to the CI group. By 2021, 73.39% of the foods belonged to the UN group, 10.34% to the PR group, 10.85% to the UP group, and 5.43% to the CI group.

Table 2 presents the average number of approved food items per school across the four procurement processes during the pre- and post-COVID-19 periods, categorized by NOVA classification, along with estimates of the differences between the two periods. In the post-COVID-19 procurement processes, there was a statistically significant increase of approximately 79% in the number of unprocessed or minimally processed foods (*p* = 0.003) and an increase of approximately 106% in processed foods (*p* = 0.0004). Conversely, a statistically significant reduction of approximately 39% was observed in the number of ultra-processed foods (*p* = 0.038).

The UN food group procured in 2019 primarily consisted of vegetables, fruits, animal-based foods, and cereals. In 2021, most of the items in this group were vegetables, fruits, and fruit pulps. On the other hand, there was a decrease in the bidding for ultra-processed products, especially salted meat, Calabrese sausage, chicken mortadella, turkey ham, and sausage, as shown in Table 1. The same items were observed in both years analyzed concerning the PR and UP food groups. Some examples include bread, cheese, and cake (made primarily from minimally processed foods and culinary ingredients) in the PR group and dairy drinks, biscuits, and hot dog buns in the UP group. As for the CI group, the 2019 procurement included soybean oil, salt, and sugar, while in 2021, only one item from this group was procured: butter.

### 3.2. Excess Critical Nutrients and Genetically Modified Ingredients in Procured Foods

Across the four procurement processes, a total of 202 items were procured. These included 67 different types of processed and ultra-processed foods. Table 3 presents the average number of types of processed and ultra-processed items procured per school that exhibited excessive levels of sodium, free sugars, total fats, saturated fats, and trans fats, as well as the presence of sweeteners and genetically modified organisms (GMOs).

A reduction in foods with excessive levels of critical nutrients was observed in the 2021 procurement processes, with statistically significant differences for foods high in free sugars (*p* = 0.0004), sweeteners (*p* < 0.0001), and trans fats (*p* = 0.045).

### 3.3. Origin of Procured Foods

An analysis of the variation in the origin of foods (local, regional, and national) over the studied years revealed a significant difference in the distribution of food origins (*p* interaction = 0.01), with an increase in the procurement of locally sourced items (*p* = 0.027) and nationally sourced items (*p* = 0.048). No statistically significant variation was observed in the procurement of regionally sourced items (Table 4).

## 4. Discussion

School Feeding (SF) is recognized as a right of every student in public primary education and is the responsibility of the state to ensure. The PNAE was founded on the premise of contributing to students’ growth and development in terms of learning, biopsychosocial aspects, academic performance, and the establishment of healthy eating habits by providing meals that meet students’ nutritional needs [3,4]. According to the program’s regulations, SF menus must be based on natural or minimally processed foods. The acquisition of certain items, such as soft drinks, cereals with additives or sweeteners, gelatin, mayonnaise, cakes with frosting, seasonings containing monosodium glutamate, and other similar products, is explicitly prohibited [4].

The results of the present study indicate an improvement in the nutritional quality of foods procured for school meal menus following the critical COVID-19 pandemic and the concurrent implementation of Resolution No. 6 in 2020, compared to procurement conducted prior to this period. This improvement is evidenced by an increase in the procurement of fresh, minimally processed, and processed foods, a reduction in the acquisition of ultra-processed foods, and a decrease in the presence of foods containing excessive amounts of free sugars, trans fats, and sweeteners, which are considered critical nutrients. We believe this aspect is the differentiating feature of our study compared to previously published studies, which have analyzed the nutrient profile of purchased foods.

The PNAE is currently regulated by Resolution No. 6, published in 2020, which introduced a series of new requirements and tightened rules previously established in other normative acts. Its provisions state that most SF menus consist of unprocessed or minimally processed foods and include a list of products prohibited from being procured. Additionally, it specifies that at least 75% of the financial resources must be allocated to purchasing unprocessed or minimally processed foods. In comparison, a maximum of 20% of the resources may be used to procure ultra-processed products [4]. Therefore, the improvement in the nutritional quality of foods procured in 2021 in the analyzed schools is understood to be influenced by the publication of Resolution No. 6 in 2020.

The COVID-19 pandemic also triggered changes in the PNAE. As part of efforts to mitigate the spread of the virus, in-person classes and several other societal activities were suspended, leading to the temporary cessation of traditional school meal distribution. To address the negative impacts of this suspension, Law 13.987/2020 was enacted, authorizing the distribution of foodstuffs to the families of students enrolled in public schools [13]. Complementing this law, Resolution 02/2020 issued by the National Fund for Educational Development (FNDE) mandated that all PNAE resources be exclusively allocated to ensure student nutrition in public schools during the suspension of classes. Furthermore, food items must be distributed in kits defined by the local nutrition team while respecting regional habits and cultural practices. Priority was to be given to procuring fresh or minimally processed foods. These kits comprised parboiled rice, cornmeal for couscous, beans, powdered milk, spaghetti, canned fish (sardines), cream cracker biscuits, salt, and sugar [14].

Bidding processes for food purchasing carried out in 2021 were impacted by the COVID-19 pandemic. During this period, the purpose of the food purchase was to provide food kits for students in federal schools. These kits were composed of sardines, crackers, rice, corn flour, and pasta, among other foods. A second bidding process was conducted for the resumption of in-person classes in the second semester of 2021. However, the constant fluctuation in food prices, combined with the lack of adjustments in the funds transferred by the government for the purchase of food in schools and the scarcity of local suppliers, resulted in the non-acquisition of various requested food items. Consequently, essential foods such as meat, dairy products, poultry, fresh fish, and vegetables were not procured at the time of the reopening of classes [15,21]. This situation underscores the need for a more in-depth analysis of the adequacy of financial resources allocated for school meals and the importance of strengthening the network of local suppliers to ensure the provision of healthy and diverse food options for students.

Studies show that meal distribution measures have been implemented on a global scale. In the United States of America (USA), Congress authorized the distribution of meals to parents and guardians of students [22,23]. Similarly, in Uruguay, meals were distributed to students, as was the case in Puerto Rico. Other countries also adopted the distribution of food kits, such as Argentina, Peru, Ecuador, Colombia, Chile, and other Latin American countries [24].

The literature reveals a lack of studies analyzing foods procured exclusively through public procurement for the PNAE. Moreover, no studies were found examining foods procured before and after the COVID-19 pandemic for the same purpose as this study. Existing research has predominantly focused on institutions that utilized public calls or similar methods or a combination of public calls and procurement as purchasing methods without any connection to the abovementioned pandemic. In a study by Nogueira et al., analyzing the nutritional profile of foods purchased through a public call, together with the bidding for menus in institutional restaurants during 2020, it was found that 60.8% of the foods had excess sodium, 40.1% had excess free sugars, and 16.2% contained the presence of sweeteners. They also presented expressive numbers regarding the excess of total fats, saturated fats, and trans fats, being 43.6%, 46.9%, and 21.3%, respectively. In addition, most foods had an excess of more than one critical nutrient. They also identified the purchase of foods with GMOs, mainly related to the acquisition of cream crackers, which is a popular food in school meals, due to its low price and high acceptance [25]. Therefore, the present study provides novel data on this topic.

Existing studies examining the relationship between COVID-19 and school meals primarily focus on food insecurity in students’ households during the pandemic and dietary changes among students during this period. Data indicate that most adolescents experienced increased consumption of more natural foods after the pandemic. A study analyzing changes in the eating habits of Brazilian adolescents during the pandemic found an increase in the consumption of fruits, vegetables, and legumes (FVL). In contrast, the consumption of ultra-processed foods remained stable [26].

In their study, Ruiz-Roso et al. (2020) investigated dietary trends among adolescents from Brazil, Italy, Spain, Chile, and Colombia during pandemic-related social isolation. The findings revealed an increase in the intake of FVL and a rise in the consumption of sweets. Brazil showed a marked increase in the consumption of fresh or minimally processed foods [27].

However, studies also reveal that despite an increase in the consumption of healthier foods, there was a rise in the consumption of ultra-processed foods, such as sweets, chocolates, fried foods, and other products. A study conducted with Brazilian adolescents reported a high incidence of ultra-processed food consumption (13.8%) and low FVL intake (20.3%) among the participants [26]. Ruiz-Roso (2020) similarly observed that, despite the increase in healthier foods, there appeared to be no improvement in the overall diet quality [27].

These findings are alarming, given that adolescence is a crucial phase characterized by significant social and psychological changes. It is also a sensitive period for the formation of new habits, including unhealthy dietary patterns. High consumption of ultra-processed foods and foods rich in critical nutrients is linked to adverse health outcomes, particularly during this life stage, which can compromise growth and development [28]. Furthermore, the consumption of ultra-processed foods is positively correlated with micronutrient deficiencies [29] and the onset of various diseases, such as insulin resistance and liver diseases, in adolescents [30].

During the pandemic, studies assessing the nutritional status of adolescents were limited, leading to a predominant focus on research addressing dietary intake and physical activity levels. Social isolation contributed to reduced physical activity among Brazilian adolescents, while increased consumption of ultra-processed foods was associated with weight gain. Studies from this period report a wide range of nutritional outcomes, from malnutrition to severe obesity, often associated with food insecurity and increased sedentary behavior [31,32,33,34].

A study analyzing the origin of foods procured by public educational institutions identified a high frequency of items sourced nationally, meaning from locations different from where they would be consumed, with the majority being ultra-processed foods. It was also observed that the procurement of nationally sourced foods occurs predominantly in institutions that utilized competitive bidding as their purchasing process, as with food procurement for federal schools in RN [25]. This can partly be explained by the economic and social disparities between production levels, particularly when comparing large industries to family farming and/or small-scale producers.

In the present study, a significant increase in the procurement of locally sourced items was observed in 2021, as shown in Table 3. This may be associated with the increase in the procurement of natural and minimally processed foods that year. Most foods within this level of processing are typically sourced from family farming or local vendors and, consequently, originate closer to the final consumption site.

Public food procurement policies, such as the PNAE, influence various aspects of the food system, from production to consumption, by regulating which foods can be purchased, from whom they can be bought, and consequently, the origin of the foods provided in SF [35]. The literature highlights that several stages of food production are responsible for greenhouse gas emissions, one of which is transportation. Thus, reducing the distance between the origin of the food and its final consumption benefits local producers and the environment [10].

Despite the existence of social programs aimed at promoting the development of family farming, such as the National Program for Strengthening Family Agriculture (PRONAF) and the Oriented and Supervised Rural Microcredit Program (AGROAMIGO), numerous challenges still hinder small-scale farmers from participating in the public procurement process [36,37].

This study is limited to presenting data from Federal High Schools in RN. The results may not reflect the precise influence of the Resolution governing the PNAE because, coincidentally to its publication, a steep increase in the inflation rate and staple food costs also occurred, which was likely to have influenced the acquisition of food products in general. However, our conclusions that the resolution resulted in the lesser acquisition of unhealthy foods still hold, as there was no decrease, and even an increase, in the acquisition of unprocessed and processed foods. On the other hand, this study was based on a representative sample, analyzing different periods of the execution of the PNAE in these institutions (before and after the COVID-19 pandemic), encompassing the entirety of the products procured during the analyzed period. Furthermore, this study examines changes in the pattern of procured foods following the implementation of the new regulation governing the PNAE, Resolution 06/2020, which introduced new requirements. These changes were reflected in the improved nutritional quality of the foods procured to compose the school meal menus in Federal High Schools in RN.

## 5. Conclusions

The analysis of food procurement by federal schools in 2019 and 2021 revealed an improvement in the quality of procured foods in 2021, with higher proportions of natural or minimally processed foods and a reduction in the procurement of foods with excessive levels of critical nutrients. Regarding the origin of the foods, the 2021 procurement process also showed an increase in the number of locally sourced items. As 2021 marked the implementation of the new PNAE Resolution, these results underscore the importance of public policy regulations in promoting healthier and more sustainable diets. This is particularly evident in the case of the PNAE, which establishes standards and limits for the quantity and quality of foods to be acquired through the program.

Additionally, it is important to acknowledge that 2021 corresponds to the post-pandemic period of COVID-19, which heightened the urgency of ensuring the quality of school meals. This period improved the nutritional quality of the procured foods, supporting the food and nutrition security guarantee for all students.

## Figures and Tables

**Table 1:** Food items approved through procurement processes in Federal Schools of Rio Grande do Norte, categorized by food group, according to the year.

Food Group	2019	2021
Cereals, Bread, and Tubers	Sweet potato, fine oatmeal flakes, cornmeal for couscous, cassava, cassava flour, spiral pasta and spaghetti, corn grits, potato, parboiled rice, polished rice, wheat flour, bread, assorted-flavored cake, canned corn, cream cracker biscuits, maize-flavored biscuits, coconut-flavored ring-shaped biscuits, hot dog bun rolls, assorted butter biscuits, oval maize-flavored biscuits, salted biscuits, and corn starch biscuits.	Polished rice, cornmeal for couscous, spaghetti, sweet potato, potato, frozen cassava, assorted-flavored cake, chocolate cake, bread, cream cracker biscuits, roll bread/buns, hot dog buns, sweet coconut bread, and breadcrumbs.
Vegetables	Tomato, garlic, white onion, cilantro, green bell pepper, carrot, Chinese cabbage, lettuce, beetroot, chives, chayote, collard greens, pumpkin, chili pepper, cabbage, green beans, cucumber, eggplant, zucchini, and tomato paste.	Fresh garlic, garlic paste, white onion, chives, carrot, cilantro, green bell pepper, tomato, pumpkin, iceberg lettuce, chayote, zucchini, Chinese cabbage, eggplant, beetroot, collard greens, cucumber, yellow bell pepper, red bell pepper, white cabbage, red cabbage, arugula, and green beans.
Fruits	Pineapple, banana, apple, watermelon, melon, various fruit pulps (acerola, cajá, cashew, guava, mango, grape, and soursop), orange, papaya, and mango.	Pineapple, banana, orange, apple, papaya (formosa), watermelon, melon, tangerine, lime, guava, various fruit pulps (acerola, cajá, cashew, guava, mango, and grape), lime, mango, and guava.
Legumes	Pinto beans, black beans, green beans, textured soy protein (meat-flavored and chicken-flavored), and soy milk.	Pinto beans and green beans.
Meat and Eggs	Chicken eggs, beef (round cut), chicken breast fillet, chicken drumstick, and thigh, whole chicken breast, fish fillet (hake), fish steak (hake), pork chop, pork loin, salted meat, Calabrese sausage, chicken mortadella, turkey ham, and sausage.	Canned fish (sardines).
Milk and Dairy Products	Powdered milk, pasteurized fluid milk, UHT fluid milk, mozzarella cheese, curd cheese, strawberry-flavored dairy drink, skimmed curd, and cream.	Powdered milk, mozzarella cheese, and strawberry-flavored dairy drink.
Oils and Fats	Margarine, soybean oil, extra virgin olive oil, and butter.	Butter.
Sweeteners and Sweets	Chocolate powder, sucralose sweetener, condensed milk, guava-flavored candy bars, and crystal sugar.	-
Herbs, Spices, and Condiments	Ground cinnamon, bay leaves, oregano, black pepper, annatto (coloring), tomato sauce, soy sauce, cocoa powder, mayonnaise, vinegar, and salt.	Refined salt.

**Table 2:** The average number of food items of publicly procured foods per federal high school in the state of Rio Grande do Norte in 2019 and 2021, by NOVA classification.

NOVA Classification	2019		2021		Difference			*p*-Value
	Mean	SD	Mean	SD	Mean	95% CI		
Unprocessed or minimally processed	16.2	13.6	28.9	11.3	12.8	4.97	20.5	**0.003**
Processed	2.30	1.53	4.70	1.45	2.40	1.24	3.56	**0.0004**
Ultra-processed	7.80	5.03	5.00	1.49	−2.80	−5.42	−0.18	**0.038**
Culinary ingredients	3.15	1.8	2.80	0.52	−0.35	−1.00	0.30	0.27

Legend: SD: standard deviation; CI: confidence interval.

**Table 3:** The average number of food items per federal high school in Rio Grande do Norte of food types procured with excessive critical nutrients, sweeteners, and GMOs in 2019 and 2021.

Critical Nutrient	2019		2021		Difference			*p*-Value
	Mean	SD	Mean	SD	Mean	95% CI		
Sodium	5.00	3.67	4.85	1.18	−0.15	−2.08	1.78	0.87
Free Sugars	1.55	0.60	0.80	0.52	−0.75	−1.12	−0.38	**0.0004**
Sweeteners	3.25	1.48	0.10	0.45	−3.15	−3.91	−2.39	**<0.0001**
Total Fats	4.15	3.20	2.75	1.07	−1.4	−3.09	0.29	0.10
Saturated Fats	5.45	3.43	4.00	1.59	−1.45	−3.43	0.53	0.14
Trans Fats	1.60	0.50	2.30	1.22	0.70	0.02	1.38	**0.045**
GMOs	2.50	2.37	2.05	0.51	−0.45	−1.58	0.68	0.42

Legend: SD: standard deviation; CI: confidence interval; GMOs: genetically modified organisms.

**Table 4:** Change in the number of procured foods by origin between the years 2019 and 2021 in Federal High Schools in Rio Grande do Norte, Brazil.

Food Origin	Difference	SD	95% CI		*p*-Value
Local	8.75	16.4	1.09	16.4	**0.027**
Regional	−0.3	0.42	−2.84	2.24	0.81
National	2.5	5.29	0.03	4.97	**0.048**

Legend: SD: standard deviation; CI: confidence interval.

## Data Availability

The data presented in this study are available in the official federal government procurement platform at https://comprasnet.gov.br/ (accessed on 1 September 2022). These data were derived from the following resources available in the public domain: 2019: http://comprasnet.gov.br/livre/Pregao/AtaEletronico.asp?co_no_uasg=158155&&uasg=158155&numprp=162019&codigoModalidade=5&Seq=1&f_lstSrp=&f_Uf=&f_numPrp=162019&f_coduasg=158155&f_codMod=5&f_tpPregao=E&f_lstICMS=&f_dtAberturaIni=&f_dtAberturaFim=&idLetra=rbfb9b&idSom=&Submit=Confirmar; 2019: http://comprasnet.gov.br/livre/Pregao/AtaEletronico.asp?co_no_uasg=158374&&uasg=158374&numprp=32019&codigoModalidade=5&Seq=1&f_lstSrp=&f_Uf=&f_numPrp=32019&f_coduasg=158374&f_codMod=5&f_tpPregao=E&f_lstICMS=&f_dtAberturaIni=&f_dtAberturaFim=; 2021: http://comprasnet.gov.br/livre/Pregao/AtaEletronico.asp?co_no_uasg=158365&&uasg=158365&numprp=22021&codigoModalidade=5&Seq=1&f_lstSrp=&f_Uf=&f_numPrp=22021&f_coduasg=158365&f_codMod=5&f_tpPregao=E&f_lstICMS=&f_dtAberturaIni=&f_dtAberturaFim=; 2021: http://comprasnet.gov.br/livre/Pregao/AtaEletronico.asp?co_no_uasg=158155&&uasg=158155&numprp=42021&codigoModalidade=5&Seq=1&f_lstSrp=&f_Uf=&f_numPrp=42021&f_coduasg=158155&f_codMod=5&f_tpPregao=E&f_lstICMS=&f_dtAberturaIni=&f_dtAberturaFim= (accessed on 1 September 2022).
